# An Anticipatory Approach to Ethico-Legal Implications of Future Neurotechnology

**DOI:** 10.1007/s11948-024-00482-4

**Published:** 2024-05-15

**Authors:** Stephen Rainey

**Affiliations:** Department of Values, Technology and Innovation (VTI), Delft University of Technology, Delft, The Netherlands

**Keywords:** Neurotechnology, Future, Law, Policy, Responsibility, Anticipation

## Abstract

This paper provides a justificatory rationale for recommending the inclusion of imagined future use cases in neurotechnology development processes, specifically for legal and policy ends. Including detailed imaginative engagement with future applications of neurotechnology can serve to connect ethical, legal, and policy issues potentially arising from the translation of brain stimulation research to the public consumer domain. Futurist scholars have for some time recommended approaches that merge creative arts with scientific development in order to theorise possible futures toward which current trends in technology development might be steered. Taking a creative, imaginative approach like this in the neurotechnology context can help move development processes beyond considerations of device functioning, safety, and compliance with existing regulation, and into an active engagement with potential future dynamics brought about by the emergence of the neurotechnology itself. Imagined scenarios can engage with potential consumer uses of devices that might come to challenge legal or policy contexts. An anticipatory, creative approach can imagine what such uses might consist in, and what they might imply. Justifying this approach also prompts a co-responsibility perspective for policymaking in technology contexts. Overall, this furnishes a mode of neurotechnology’s emergence that can avoid crises of confidence in terms of ethico-legal issues, and promote policy responses balanced between knowledge, values, protected innovation potential, and regulatory safeguards.

## Introduction

This paper explores the scope for using imagined future scenarios to prompt useful ethico-legal reflections for emerging consumer neurotechnologies. The device of imagined future scenarios is well known as a method especially relating to ‘science fiction prototyping’ (*Creative Science Foundation– CSf– A partnership with you to create the future*, no date). Futurist scholars have recommended approaches that merge creative arts with scientific development in order to theorise possible futures toward which current trends in technology development might be steered (Johnson, 2011, 2013). Taking a creative, imaginative approach like this in a neurotechnology context can help move development processes beyond considerations of device functioning, safety, and compliance with existing regulation, and into an active engagement with potential future dynamics brought about by the emergence of the neurotechnology itself in specific contexts. While some empirical work has suggested exposure to science fiction can boost the acceptability of neurotechnology devices (e.g. Koverola et al., 2022; Grinschgl et al., 2023) this article seeks to highlight how this kind of exposure can be specifically useful in ethico-legal senses. The aim isn’t to recommend ways to boost acceptability, but to explore how future scenarios can steer not just technology innovation trajectories, but specifically legal and policy environments.

Critics of imagined scenarios in this kind of context warn of a slide into an overly speculative mode of thinking, that can serve to distract from already-existing ethical issues through drawing on resources better spent in tackling the here and now, or even undermining the authority of neuroethics as an approach (Gilbert & Goddard, 2014). Other critical voices meanwhile caution that various measures ought to be taken to finesse the use of speculation in order that it doesn’t produce unwanted outcomes, for example by being clear about assumptions, and exercising ‘cautious skepticism’ about projecting possible futures (Outram, 2012; Racine et al., 2014). Holding these fair comments in mind, in this article the species of speculation employed is self-consciously not that critiqued. Poor uses of speculation are based in pure fiction and can have the effects anticipated in the literature. Instead, here, a form of constrained speculation about possible advances and applications is recommended. This is based in reasonable extrapolations from current technological realities, ambitions, trends, and projections based in contexts of likely development (e.g. privately financed direct-to-consumer neurotechnologies, developed without reliable ethical oversight, hungry for data, and marketed with voluminous hype). The questions raised are therefore less concerned with ‘what would we do if…’ and more centred on, ‘if the kinds of things highlighted in these scenarios could happen, what should we think about it, and how could we prepare for or mitigate it?’. The imagined scenarios aren’t to stand for probable futures, but reflective jumping-off points, from which to assess the here and now, especially in terms of preparedness for possible futures.

Imagined scenarios can engage with potential consumer uses of devices that might come to challenge existing regulations and/or other conventions, as with the Asilomar conference of 2010 (Vlek et al., 2012). An anticipatory, creative approach can imagine what such uses might consist in, and what they might imply. This is motivated by the idea that, in cases of complex emerging technologies like neuromodulation devices, no single set of interested parties might be best placed to guess at all the possible ethical and legal questions that could arise from those devices in use. Below, two general outlines for potential future implications from consumer neurotechnology are developed, each with ethico-legal implications. The first scenario imagines a court case centring on BCI use while driving, the second on questions for policy raised by neurotechnology and data use. These are not fully-fledged attempts to engage in sci-fi prototyping, it should be noted, but rather are efforts to demonstrate how imaginative engagement with current technologies in the vein of that method can anticipate interesting ethical and legal questions. The central aim is not to steer the technology itself towards some desired end. Here the value of imagining emerging technologies *in context* in order to test, through constrained speculation, what kinds of ethical and legal norms might be tested by the technology’s presence. The aim is not to finesse the technology, but to pre-empt potential ethical and legal gaps in order to complement technological development with contextual preparedness.

### Current Consumer BCI Development Context

The mechanism of many existing consumer BCIs is to provide neurofeedback, in order to allow users to gain insight on and try to modify intentionally, their own brain activity (See for instance Sitaram et al., 2017). Some of the goals included in marketing include using devices like this to produce meditative states, or improved focus (*Muse - Meditation Made Easy*, no date). While these devices do raise ethical issues, the main questions hanging over them are about their efficacy (Coates McCall et al., 2019). Whether they ‘work’ or not is often assessed according to user satisfaction, rather than anything specifically rooted in the technology’s functioning. What’s more, literature suggests consumers are not alone in being apt to believe dubious claims couched in neuroscientific terms (Beyerstein, 1990; McCabe & Castel, 2008; Rhodes et al., 2014). This suggests consumer vulnerability to neurohype, likely compounded by predominantly positive media coverage (Gilbert et al., 2019).

A future iteration of consumer neurotechnology for the market will likely include devices that directly, perhaps automatically, influence neural activity with the goal of modifying mental states or dispositions thereby (Brown et al., 2016; Leite et al., 2017; Rainey, 2022). These devices might be expected to be more verifiably effective in modifying brain activity. The nature of such devices as ‘closed loops’– both recording and stimulating brain activity– would include evidence of influencing brain states in that they will apply modulatory outputs like electrical or magnetic fields to the brains of users. Whether or not the effects produced would correlate with a user’s increased control over mental states would be a further question in need of examination.

Based in their functioning, and the verifiability of neuromodulatory effects, future devices will be more easily seen as effective at changing the function of the brain. To pre-empt what this might mean for users, the likely emergence of ethical and legal issues for such devices aimed at consumer markets can be imagined. Toward that end, two broad scenarios are imagined below.^1^ These scenarios are intended as illustrative of a kind of approach that ought to be included in neuromodulatory device development aimed at consumer uses. They make vivid some questions relating to the efficacy of future consumer neurotechnology. As an exercise in anticipating future technological change, the scenarios developed will frame likely concerns that ought to prompt legal or policy responses.

To develop the illustrative scenarios, a future of forty years from now is to be imagined. In this future, the consumer neurotechnology market has been growing and attracting a lot of positive attention from consumers. The general public are excited by this novel technology, and its promise of greater control over brain states. Using the technology, customers hope to control their mental states and dispositions. These neuromodulation devices can stimulate brain areas to be more active, and dampen others that are overactive, in order to meet users’ desires. Despite some scepticism in expert circles about the effectiveness of BCI devices across these contexts, the marketing is confident, and consumers are satisfied with their devices. Regulators have taken a light touch approach, eager not to stifle the emerging neurotechnology industry and consumer market. The hands-off regulatory approach, however, is set to encounter a challenge, as a high-profile court case begins: that of Smith vs. Jones and a car accident.

### Scenario 1

In Smith and Jones’ car accident, each driver was wearing a neuromodulation BCI. Each claims the other was responsible for the accident. Smith claims he ought not to be considered liable for the accident because he was wearing his BCI and was therefore verifiably in a state of heightened concentration, and therefore was taking extra care in driving. Jones claims he should not be liable for the accident because he was wearing his BCI and was therefore not in full control of his actions. On the one hand, Smith wishes to claim his BCI made him an extra safe driver so he could not be to blame. On the other, Jones claims he can’t be considered as fully in control because he was under the influence of his device.

Smith’s point is that BCI technology has made genuine claims about boosting users’ concentration, and that he believes these claims, in good faith. Smith considers himself to be acting as a particularly virtuous driver by taking care to maximise his concentration levels by neurotechnological means. Jones’ point is that the BCI technology was marketed as being able to genuinely and significantly influence brain activity. This means, from his point of view, that he can’t be fully responsible for what he does when using it. The device, as far as Jones is concerned, manifests a case of hybrid control: he was not solely responsible for his actions, since his device was actively influencing his brain states. He thinks that his responsibility ought to be considered as diminished in proportion with this lower level of control. BCI manufacturers are involved in the case as witnesses: they must defend claims made about their products effectiveness for Smith’s side of the argument. But in doing so, they risk substantiating Jones’ argument and suggesting that their devices are risky to use. Regulators too are concerned that their approach to neurotechnology may be questioned.

If Smith wins the case, a precedent would be set regarding the enhancement possibilities of neurotechnology– one conclusion to this could be that it became seen as irresponsible *not* to use BCI devices. Ought policymakers, in this case, to make their use compulsory? But if Jones wins the case, regulators could be criticised for allowing unsafe technology to be on the market. Regulation might need to be sharpened up, leaving lots of BCI device owners unnerved.

### Discussion of Scenario 1

This scenario imagined a neuromodulatory device in use, and a possible problem with responsibility and action emerging in a legal context. Discussions about *moral* responsibility with respect to neurotechnology use sometimes focus on the hybrid nature of control produced when such devices are used, contrasting with standard cases of responsibility and control as discussed by Fischer and Ravizza (1998). The hybrid nature of control in neurotechnology contexts may produce a ‘responsibility gap’, meaning users of such technology ought not to be considered as responsible as they might have been without use of the device (Matthias, 2004).

*Legal* responsibility is typically considered as consisting of *mens rea* and *actus rea*, meaning an intention to commit an act (a mental state) and the commission of the act (physical action) are required in order to ascribe legal responsibility. In some cases of neurotechnology mediated action, these can appear to come apart. The physical act in particular can be missing, for instance where someone in a total state of paralysis operates a device using their brain activity alone (Bublitz et al., 2019). In these cases, the user makes no physical movement at all– they cannot– yet they are active in the world through technological means. This creates puzzling questions about whether a person in these unusual circumstances could be considered legally responsible at all for their ‘actions’. The literature in these areas is lively. But the imagined case of Smith and Jones’ closed loop, neuromodulatory technology might pose a different sort of question.

In the case of Smith and Jones, and the car accident, there isn’t really a question of *mens rea*– neither party *intended* to have a car accident– that’s part of what it is to have an accident. While carelessness or negligence are potentially *mens rea* for the purposes of criminal culpability, this is not clearly the situation here. Indeed, this might be part of what is at issue in the case. This is one element of interest in the case, since it involves a contextualised use of a device the evaluation of which could have ramifications for future regulation of and claims about the very technology.^2^ Actions are not being mediated by neurotechnological means, as in a case of a BCI-controlled limb but rather, the brain itself is the target of the action.

Rather than an instrumental intervention in the wider physical world by technological means, the instrumental intervention is on the user’s own neural state. After an initial decision to use the device, moreover, the closed-loop nature of the device means control over the neural intervention is not hybrid, but automatic. The user does not split control over the realisation of target brain states with the system but gives over control. The initial decision to use the device includes the intention to realise a mental state of heightened concentration. If the device does indeed realise this mental state, that initial decision is successful. If not, the decision is unsuccessful. But in either outcome, the user themselves might not be straightforwardly held ‘responsible’ for the mental state that is realised. After the initial decision the user has no role in, say, the timing or intensity of neural stimulation produced by their device. The user *undergoes* the activity of the device once it is underway. Whether or not this would be judged to constitute carelessness or negligence would have far-reaching consequences for the neurotechnology development sector.

If the device really does create mental states through its activity, then it is difficult to see how the actual mental states that are realised are the responsibility of the device users. Actions taken while using the devices would apparently arise from an altered mental state not the direct responsibility of the user. Might this spell trouble for assigning legal responsibility for actions for users of closed loop neuromodulatory devices? The question from Smith and Jones might not be ‘who is responsible for the accident,’ so much as ‘can the law adequately account for novel, neurotechnologically produced mental states?’ This echoes some discussion of free will enhancing drugs in in Glannon (2011) and how responsibility might be affected in scenarios where agents have increased capacity in that regard (Vincent, 2013). An issue pertinent here, derived from that discussion, could centre on whether and how ‘super’ concentrators using neurotechnological devices ought to be ‘super’ punished for errors given their enhanced capacity.^3^ Again, this would call for reflection not just on applicability of laws in a ‘what if?’ scenario, but on whether the appropriate concepts of responsibility existed in law, given the novel technology.

An account of legal responsibility that could accommodate the neurotechnological creation of mental states would have to consider whether or not the foreseeability of the desired outcomes of neuromodulatory technology use was sufficient to warrant a user’s confident use of that technology. If, in thinking his device would boost his concentration, Smith was warranted in higher confidence in his driving then he would seem to be virtuous in driving under the influence of his device. This would require that there was a standard by which to evaluate neuromodulatory devices’ effectiveness. The importance of such a standard is highlighted by Jones’ insistence that he ought not to be held responsible owing to the use of the very same device as Smith. His claim is that because he had the accident, this suggests the absence of heightened concentration. But since he was not in control of the device, and by hypothesis thereby not in control of his own pro-concentration mental states, he ought not to be held responsible. The arguments of Smith and Jones are symmetrical with respect to the nature and operation of the neuromodulatory device, but divergent in the evaluation of the mental state produced. The mental state they do share is that leading to the initial decision to use the device in the first place– they each intended to improve their concentration to make them better drivers.

Something like concentration isn’t a binary state, of course, and comes in degrees. If Smith’s faith in his device meant he expected the device to do his concentrating for him, he might become reckless or negligent and be the cause of the accident. Similarly, if Jones had so little faith in his ability to exert control while using his device, we might reasonably question his wisdom in using it at all. In terms of the responsibility question we might expect degrees there too: Smith and Jones each might be thought of as aiming to be more responsible in driving through using their devices. Smith’s conclusion is that this aim absolves him of responsibility for the accident– the responsibility of the aim ‘carries’ through to the actions leading from it. Jones’ point is that the aim, however well-conceived, was apparently thwarted and so he ought to be absolved of responsibility. If Smith was wrong to have such confidence, but based it in claims made by technology developers, he might reasonably claim diminished responsibility in virtue of the neurohype he believed in good faith. In this instance, regulators would have to answer for their lax approach that permitted poor information to filter into the consumer neurotechnology market. If Jones was wrong to suggest his device failed, and that it did in fact boost his concentration to some appreciable extent, he might switch his defence to that of Smith. Here again the foreseeability of the neuromodulatory device’s effects is of central importance.

These kinds of reflections warrant inclusion in neurotechnological development as they include future uses, beyond standard considerations of device effectiveness, safety, regulatory conformity etc. The development of a wider, imaginative standard by which to evaluate consumer neurotechnological effectiveness would be a clear opportunity for interdisciplinary engagement between (at least) philosophy, neuroscience, consumers, and the law. A fruitful dialogue among these parties, would then be able to feed into regulatory and policy discourse in order to frame evidence-led, and well analysed responses to realistic prospects presented by emerging technologies. Smith and Jones (or similarly framed scenarios)^4^ raise deep questions linked to the very presence on the consumer market of neuromodulatory devices. Including detailed imaginative scenarios as part of the technology development could include the deflation of neurohype which would not only benefit regulation, but also the consumer market. If the reliable production of identifiable mental states by means of consumer-grade neuromodulatory devices is not deemed feasible, for instance, based in evaluation by a multi-disciplinary group, regulators would be empowered to constrain claims made about such technology (as they do already for medical claims). The scenarios, in this sense, don’t need to be accurate predictions for a concrete future context, but can serve their purpose in raising good questions. Indeed, the aim isn’t to predict the future, but to imaginatively engage with potential future impacts in order to boost reflection in technology development.

The second scenario occurs in the same broad future context as this first one but examines technology providers’ roles more than that of users, raising policy questions. In this case, a user comes to feel somewhat cheated and misled through having used their device while unaware of a larger context of its operation.

### Scenario 2

Just like a heart patient might be prescribed a pacemaker for a heart rhythm issue, it was recommended by her doctor that Ada use a neuromodulator, following diagnosis with a stress disorder. She knows others who use similar devices for other conditions, including some friends with attention deficit syndrome who use their devices to promote states of increased focus. Recently, the devices have been upgraded, so they no longer need to be manually controlled. They can now run on ‘automatic,’ relieving the user of the burdens of self-monitoring. Ada has been relying a lot on her neuromodulator because of this, using it to fine tune her brain activity to help keep out overwhelming stress, while not numbing herself to the urgency of her task.

The simply-stated aims of this neuromodulatory device– which works as advertised– conceal a large data ecosystem behind the scenes. This neuromodulatory device is an affective neurostimulator which detects brain activity characteristic of disordered emotional experience and delivers stimulation to dampen unwanted emotional responses. From the user perspective, it is a useful mood enhancer. The devices record brain activity as it happens, across the user’s whole brain, using EEG electrodes in some discreet headgear. The activity is compared with exemplars of ‘normal’ brain activity stored on a cloud platform. When the activity being recorded starts to depart too much from normal, the headgear receives a signal, and the electrodes emit electromagnetic stimulation to bring local brain activity back towards a normal state. The device software includes information on users’ mental health, so that it can respond to the kinds of brain activity characteristic of conditions like chronic anxiety or ADHD.

One day Ada is reading a newspaper article and notices a story about the recent financial growth experienced by the manufacturers of her device, including its partnerships with marketing and security industries. She is shocked and feels exposed. The benefits of her device to her seem undeniable. But she feels like the use of her brain recordings with such partners should never have been possible. It feels grotesque to her. But she has benefitted from the overall market in her brain recordings. She does not know what to do. Her options seem to be to ditch her device in protest, or to continue using it despite feeling exploited. Overall, she wishes the market in data had never been allowed to open– or that she had never found out.

Ada and the other users she knows are very happy with the system they use. It gives them a greater sense of being liberated from sometimes debilitating anxiety and agitation. But as users, they are not very well informed about exactly how their devices operate. This raises questions about neurotechnology companies in terms of the brain data they generate and process and how informed the use of neuromodulatory devices ought to be.

### Discussion of Scenario 2

Part of the appeal of a device like that used by Ada comes from its easy-to-use format. This ease of use is rooted in data processing. The accuracy of the data in the cloud relies on huge amounts of data being collected from many devices and stored online (Kellmeyer, 2018). The vast amount of aggregated data is processed according to complex algorithmic approaches, from which are developed general pictures of ‘brain activity’ for different user aims. For Ada, this means she can rely on her system to alleviate anxious states.

More generally, besides device users, these huge stores of data will attract interest from a variety of interested parties, including ‘big tech’. Marketing companies want to know about the brain states users manifest under different kinds of conditions so that they can target their ads more effectively. The ‘neuromarketing’ industry is growing, fuelled by insights gained from these kinds of devices (Spence, 2019). Users might not often think about it, but the ability their devices have to automatically detect and modify brain activity relies upon access to the cloud data, which means they are always locatable when their devices are on automatic mode. For marketing companies, this could mean ubiquitous brain recording complete with location data that allows a combined neuro-geographical picture of users’ everyday life. The companies that would develop the devices’ subsequent profits allow for more product innovation, which in turn permits greater data accuracy, creating an ever-evolving brain data market.

From a neurotechnology company point of view, in order to continue to be able to operate, innovate, and remain financially viable, it is important to be able to realise and monetise assets. The neurotechnology company behind Ada’s device sits on a trove of brain data, without which they could not provide the services she has come to value. With an aim to continue to provide and improve these services, and to provide more besides, the company sees the granting of access to their data trove as a good investment to make these beneficial therapeutic interventions sustainable. Part of how the devices function for *any* user is through the processing of *every* user’s brain data. The aggregation of large amounts of data is what helps create the accurate models used in prompting stimulation that alters brain states. When they grant access to their data stores, they are not thereby exposing the identity or health condition of any given user. Nevertheless, for a user like Ada the use of data by third parties feels like the device has changed in nature. Over and above a neuromodulatory device to aid a stress disorder, it now feels like a data collection tool for marketing agencies and big tech companies.

Assuming Ada would not be alone in responding negatively to the diversification of uses of her brain data in providing non-therapeutic modelling resources, this points to a potential schism between neurotechnology developers and users. There is no essential way to define what the device that Ada uses ultimately is– a therapeutic intervention or a neuro-social data collector. It is both. But unease attaches to the slide in emphasis from one end of the spectrum to the other. In a sense, Ada is buying access to good improved mental health through providing brain data to the highest bidder. But without a way to opt out, this could easily feel to some degree coerced. The neurotechnology developing company may think that the cost of providing this data to third parties is negligible for the outcome of mental health. But this can be seen as reversible: for the user, the cost of risking a deterioration in mental health is too high to genuinely permit withdrawal from the arrangement that markets their brain data for third parties.

Companies who market neurotechnologies have responsibilities to inform users about the kinds of expectations they ought to have concerning their devices. But brain data (currently) sits in a grey area in terms of personal data. In itself, it is not clear that brain data enjoys protections under data protection regimes like that of the European General Data Protection Regulation (GDPR), for instance, as in that regulation data is at least partly classified according to the purpose for its collection (Rainey et al., 2020). Unless Ada had been *prescribed* the use of her neuromodulator, thereby making its use a medical application, it isn’t clear what the data generated in its use would be seen as, legally. In combination with location data, she would be identifiable, and so this combined data would count as personal data. But taken on its own, brain data isn’t something from which a person can be identified, and so it may not be due any particular protection. Nevertheless, it is derived from the unique activity of Ada’s brain and is used to create a powerful neurotechnology platform which is to the benefit of many users’ mental health, as well as the profit margins of the neurotechnology companies behind it.

From a user perspective, the neuromodulator might seem to simply read brain activity and intervene when it gets out of control. But with a more detailed picture of how its interventions work, users might come to think differently about it– they might come to think about the transactions in data that underlie the function they benefit from, and how they benefit others (Rainey et al., 2019; Ienca et al., 2022). The question would be: how much information on function would be necessary to explain to device users. Too little, and some users might reasonably be considered underinformed, raising challenges for informed decision-making and consent to use devices. Too much, and users might be overwhelmed or put off using devices that would provide them with help. What’s more, where the system is complex a detailed explanation of how it works might serve as much to obscure matters for users without detailed grasp of a wider neurotechnology ecosystem.

To address these data concerns, neurotechnology companies could follow the example of European data authorities on website tracking and provide a list of all the destinations for user data, as seen in cookie notices. If a user receives a list of the companies and entities likely to receive or process their data, they can insulate themselves against surprises. Decisions to go ahead and use the devices having seen such a list could be seen as more deliberate than simply signing something like a terms of service agreement. In this case, users could decide whether those who would receive data were acceptable parties with whom to deal in data. It is probably fair to say that cookie notices on websites are not universally loved, however, and for many users these might simply become another step to get past without much thought.

Alternatively, data in neurotechnologies could be classified according to the uses it will have, not its purpose in collection. If an instance of general brain recording, for no specific purpose, goes on to generate data that is to be used in the alleviation of a diagnosed mental health disorder, that data could be classified as *medical data*. Given a medical purpose, the data would enjoy enhanced protections under data protection regimes. Specific permissions might then be required from users when instances of brain data generated from their brain activity might find its way into medical applications. This would represent a boost to data transparency for users, albeit at the cost of increased technical complexity for companies in classifying data. In terms of user experience, a line would be drawn between medical and ‘wellness’ or other recreational neurotechnology applications.

## Discussion

As with response the to the first scenario, in the second again the value of multidisciplinary dialogue is clear. Technical expertise would be required in order to ascertain what data is held where, and how it might be used, processed, or reprocessed. Data scientists, engineers, and technicians would need to be consulted. The neuroscientific or medical significance of data would also be a necessary part of understanding the scope for medical applications (and hence whether brain data ought to be considered medical data or not). Neuroscientists and medics would need to be consulted for this dimension. Philosophers might usefully analyse the representational potential of brain data, as part of a means of evaluating its value-status. Patient groups might be consulted regarding patient priorities for specific disorders, as these would represent a significant user group. With an aim of modifying policy and the regulatory environment, law and policymakers too would have to be included in order to translate the dialogue into effective policy.

The mode of multidisciplinary dialogue would boost the legitimacy of policy input through including relevant points of view and expertise, while the translation would bring effectiveness (Habermas, 1980). Crucially, it would be contextualised by means of the scenarios themselves, rather than as random imaginings, or abstract speculation. In order to imagine a more general way in which to implement this imaginative contextualising approach it will be useful now to make some of the general takeaways clearer, and justify a mode of institutionalisation for this future-facing and imaginative approach to anticipating ethico-legal issues arising from emerging neurotechnologies. This will add *feasibility* to its merits.

Part of the rationale of this discussion has been to justify the use of imagined scenarios in neurotechnology development processes specifically with respect to future legal or policy issues. This has included showing how such an approach can pre-empt detailed future ethico-legal questions in order to open space for their discussion in the present. The two main themes have come in terms of responsibility. In each scenario a variety of issues emerged relating to:

- how neurotechnologies can be presented to users.
  - attention paid to neurohype.
- the contexts in which neurotechnologies operate.
  - the brain data ecosystem required for operation.

The suggestion has been that multidisciplinarity is important here. This is not a novel suggestion in itself, but the justification here has been that such interdisciplinarity is needed to connect reflective technology development with policymaking, not only device development itself. It is also to accommodate different perspectives from different fields that can be brought into contact in order to weigh up perhaps competing values regarding technology development. As such, this represents a value sensitive approach to steering technology development (Friedman & Hendry, 2019). Multi-disciplinarity is required from a knowledge perspective since no one set of relevant parties in scenarios like 1 and 2 might be said to have all the facts. Nevertheless, each is a vital player. There is also a moral justification for multidisciplinarity in that no one group ought to be left with the responsibility for such complex decision-making.

Besides awareness that knowledge and values ought to be reflected in the ways emerging neurotechnologies actually do emerge, it is important to retain dynamism especially since some of these technologies (e.g., neuromodulation devices) might be expected to change how some people think of and value things like ‘states of mind’. With a set of complex and potentially competing values, and differing levels and types of knowledge, dialogue represents a means of gaining decision-making power over potential complexity. Without domination of the discourse by any specific participant, there is scope for value change to be reflected in considerations (Poel, 2020). How policy ought to respond to novel accounts of responsibility-taking in societies with widespread neuromodulation device use will depend on facts and values, as well as shifts in these areas. No one party or group is capable of (or morally justified in) taking responsibility for a decision affecting all. All parties ought to seen as *co-responsible* therefore for the potential directions approved or decried by answers to difficult technology-derived ethico-legal questions (Apel, 1993; Von Schomberg, 2020).

Co-responsibility is contrasted with personal, role responsibility and with collective responsibility. Personal or role responsibility is inadequate in accounting for actions in this complex sphere of technology development as the outcomes in such a sphere cannot be traced back to individual intentions to act in given ways (Fischer & Tognazzini, 2009). Action in technology development is complex and not within any specific individual’s purview. Collective responsibility is inadequate too in that the complexity of technology development includes cross-disciplinary, as well as socio-political, dimensions. No coherent collective might be identified across different stages of technology development (Apel; Von Schomberg ibid.). Co-responsibility seeks to encapsulate the idea that, individually, all relevant parties ought to be responsive to the varieties of questions that can attend all elements of technology development. The suggestion here is that co-responsibility among groups of interested parties can be taken through institutionalising multidisciplinary discourses within neurotechnological development that include connections with policymakers, whose remit connects with socio-political values. These discourses amount to endeavours to understand the potential for technology developments to influence different areas, inside or outside its stated aims. Insofar as technologies impact upon dimensions of society (e.g., legal responsibility, the costs of good mental health) it is incumbent to consider that impact carefully from multiple points of view.

The sketch of a solution to questions arising from scenarios 1 and 2 can be seen recommending an ethics of co-responsibility that goes beyond established codes of ethics and responsible innovation, and steps into territory sketched in the 2009 ‘Lund Declaration’. This declaration recommends that research ought not to simply avoid being unethical, but actively seek innovation with ‘the right impacts,’ in addressing societal challenges:

…instead of an exclusive focus on the risks and (ethical) constraints of new technologies, the question of directing or redirecting research and innovation towards societally desirable ends must be given importance in research and innovation programmes. This implies that we not only have to have professional bodies for risk assessment but also professional bodies that should look into the type of outcomes we want to get out of research and innovation processes, and the establishment of governance mechanisms that should give some direction to– or steer– the innovation process. (Von Schomberg, 2020, p. 8)

Co-responsibility requires a collective understanding of the intentions behind, aims of, and potential practices utilising technologies. But it also requires the recognition that these constitute one trajectory among a variety of others enabled by evolving research endeavours and social perspectives. Dialogue among relevant parties, not simply communication from one party to another, is necessary to gain a full picture of the potential impacts of technology arising from research. This is why a kind of research-policy infrastructure is needed that includes imaginative future scenarios. Such scenarios ought to appear in a forum at the interfaces of research and policymaking, research funding bodies, researchers, and potential end-users such that emerging findings can be anticipated, and thereby frame emerging policy discourse and future research funding priorities. In this context, technology issues can be anticipated and considered before they arise in consumer contexts.

## Conclusions

Research contexts are complex and interdisciplinary, and individual researchers can struggle to identify their own responsibility for perhaps remote effects from highly focussed research tasks they undertake. Where basic research leads to technological innovation, this can be the case. Claims that can be made about neurotechnologies of the future exemplify this, where impacts might include neuromodulation devices and mental interventions. A broad mechanism of accounting for future questions is required that can include the breadth of research activity and technological outcomes. This is true for the specifics of technology development (i.e., innovation trajectories), but also for the interfaces where technology meets the wider world in its uses. Such uses cannot reliably be determined in advance by concentrating on the nature of devices, but they can be imagined. Such an anticipatory approach offers opportunities such that policymaking can keep abreast of current technology developments, with an eye on the future. In a context of co-responsibility, research, technology development, and policy can keep pace through imaginative means. Through dialogue they can influence one another. Imaginative scenarios can provide this interactive dialogical encounter.

In a context of hyper-specialisation, in which policymakers face the challenges of fast-paced technological advance among complex societies, the institutionalisation of co-responsibility would serve to make more efficient and legitimate technology policy. This includes emerging neurotechnologies. In the case of neuroscience and neurotechnology development, it is important that those in the field recognise their role as extending beyond the lab. The neuroscientific voice is an essential one in order to evaluate claims of, for instance, neurotechnological affective control. But vital too is the willingness to see neuroscience and neurotechnology as they appear to the non-expert eye. Receptivity to imaginative challenges from beyond single disciplines is as important as clear, expert input where the stakes include handling ethico-legal questions for emerging technologies. Again, imaginative scenarios can provide the means for substantiating this co-responsibility approach.

## Footnotes

1 These scenarios are based on ideas originally brought up in a European-funded project ‘Techethos,’ under EC grant agreement 101,006,249, exploring future technology ethics issues. Different versions of the scenarios appear in that project’s Deliverable 3.6, “Evolution of advanced TechEthos scenarios.”

2 To emphasise, this is a truncated account of liability dynamics. Clearly, further, different discussions with different upshots are possible and desirable across an array of imaginable circumstances.

3 Many thanks to an anonymous reviewer for succinctly highlighting this helpful parallel.

4 The limits on imaginative scenarios might seem to be simply those of imagination itself, but of course while there are an indeterminate number of possibilities for scenarios those suggested here to be of use are far from randomly generated. They respond to technical capacity, likely innovation trajectories, regulatory reality, social and ethical values, and so on.
